# Comparison of the effectiveness of novel intervention on restricted range of motion of shoulder in young healthy subjects

**DOI:** 10.12669/pjms.37.5.3465

**Published:** 2021

**Authors:** Keramat Ullah Keramat, M. Naveed Babur

**Affiliations:** 1 Keramat Ullah Keramat M.Sc. (Sport and exercises) Helping Hand Institute of Rehabilitation Sciences, Datta Morr, Mansehra, KPK, Pakistan; 2 Prof. Dr. M. Naveed Babur (Ph.D.) Principal, ISRA Institute of Rehabilitation Sciences, ISRA University, Islamabad, Pakistan

**Keywords:** Manual therapy, Posterior capsular stretch, Prevention of injuries, Rehabilitation, Serratus anterior stretch, Sports injuries

## Abstract

**Objective:**

To evaluate the effectiveness of four novel and pragmatic interventions on the restricted range of motion (ROM) of shoulder joint in healthy subjects.

**Methods:**

The study was conducted at Helping Hand Institute of Rehabilitation Sciences, Mansehra, in 6-months duration. This quasi-experimental study recruited 120 young subjects with an equal proportion of males and females for four novel intervention groups (n=30 each group) including pragmatic posterior capsular stretch, Serratus anterior stretch, rotator cuff facilitation and acromioclavicular joint mobilization. Study variables included measurement of Reaching up behind the back (RUBTB), Reaching down behind the neck (RDBTN), and shoulder range of motion (flexion, abduction, external rotation, internal rotation). The paired t-test was used for the change in pretest and posttest variables and the Kruskal Wallis test was used to compare the change in each group of interventions.

**Results:**

All the variables improved significantly (p< 0.05) from their baseline scores for the interventions. The mean difference among the intervention groups for the variables was statistically significant (p<0.001) on the Kruskal Wallis test. Pragmatic posterior capsular stretch (PPCS) and serratus anterior stretch (SAS) improved the RUBTB and RDBTN more than the other interventions. Rotator cuff facilitation (RFC) improved shoulder rotation more than the rest of the intervention. Acromioclavicular joint mobilization (ACJM) was effective in improving the abduction and flexion ROM.

**Conclusion:**

Pragmatic interventions are effective in improving shoulder ROM in young healthy adults and recommended for the trials on prevention and rehabilitation of shoulder pathologies.

## INTRODUCTION

Shoulder pain is one of the commonest musculoskeletal complaints in people with sedentary life style^1^ and overhead athletes.^2^ Long working hours, stress and inappropriate posture are major risk factors identified in people with sedentary lifestyle.^3^ Stoop sitting while working on a computer or reading a book- or any occupation, which involves similar stoop posture makes the individual susceptible to shoulder pain syndrome.^1^,^4^ The position of the scapula in a stooped posture is protracted and changes occur in the posterior capsule, serratus anterior, acromioclavicular joint (ACJ) and rotator cuff.^5^ Similar changes in shoulders of overhead athletes occur due poor posture, stressful environment, repeated stress and overload to the posterior capsule.^6^,^7^

Gleno-humeral internal rotation deficits (GIRD) refer to the deficits in internal rotation of the dominant arm compared to the non-dominant shoulder in overhead athletes and are not uncommon in healthy university students.^8^,^9^ The repeated stress generated by the high-velocity forces against the posterior capsule stimulated the negative feedback system in a throwing shoulder and, as a result, the posterior capsule gets hypertrophied and tightness follows. The tightness in capsule not only affects the functional movement of hand behind the neck and back but also the shoulder range of motion.^10^ The tightness in posterior capsule has been clinically observed and surgically explored.^11^,^12^

Changes in structures around the shoulder joint occur both in persons with a sedentary lifestyle and overhead athletes.^9^ These are asymptomatic initially and gradually become symptomatic^2^,^5^-leading to so-called condition of subacromial impingement. A 43% increased risk of developing shoulder pain in 2 years with GIRD has been reported^7^.

To loosen and lengthen the posterior capsule, crossbody stretch, sleeper’s stretch and modified stretches are used.^13^-^15^ Serratus anterior is closely associated with scapular dyskinesis and stretching for its middle and lower-division has been recommended to optimize the function of the shoulder joint.^5^,^16^ Similarly, mobility of the thoracic spine has been linked with mobility of the cervical spine and shoulder girdle, and improved mobility of the thoracic spine has been shown with the improvement of shoulder^17^ and neck function.^18^

Posterior tilting of the scapula is primarily dependent upon motion^19^. The stiffness of the joint due to non-use in sedentary lifestyle or because of chronic pain, contribute to the altered biomechanics of the scapula motion. Scapular movement requires a mobile ACJ, especially above 90 degree^20^, but unfortunately, due consideration is not given to study its role during shoulder pathology in terms of manual therapy interventions. ACJ mobilization is therefore required to restore normal biomechanics of the scapula.

A combination of exercises and manual therapy is recommended to treat the painful conditions of the shoulder with varying success.^21^,^22^ The growing shred of evidence regarding the aetiology of indirect shoulder pathology are seldom incorporated into manual therapy intervention. Four novel interventions based on emerging evidence are devised which are expected to improve the shoulder range of motion. The purpose of this study is therefore to evaluate the immediate effects of each interventions and compare its effects on the functional movements and shoulder range of motion.

## METHODS

### Study design

A pretest-posttest design was used for each of the four intervention groups. A convenient sample of 30 undergraduate healthy students for each group was included Healthy subjects were chosen since the interventions are directed to the shoulder for range of motion and not for pain, which interfere and limit the range of motion in subjects with shoulder pathology. Inclusion criteria were scores of Grade-I and Grade-II on the shoulder mobility test of functional movement screening (FMS^R^)^23^, which reflect posterior capsular tightness and restriction of shoulder ROM. The sample size for each of the intervention was estimated from the trial of similar design with similar variables.^24^,^25^ The total sample size was therefore 120 healthy subjects. Subjects with any pathology of the shoulder and grade zero and three on the same scale were excluded since there was pain on movement or there was no restriction in the shoulder ROM respectively. The study design was approved by the Research and Ethics Committee of Helping Hand Institute of Rehabilitation Centre through a registration number REC-078 dated 2^nd^ February. Each subject signed an approved consent form. Trial were registered prospectively on clinical trial.gov (NCT04242888).

### Data collection and instruments

Shoulder mobility test of FMS was used for assessing the functional movement of the hand reaching up behind the back and hand reaching down behind the neck.^23^ A digital inclinometer (clinometer) installed on a Samsung note-8 was used for measuring the shoulder internal rotation (IR), External rotation (ER), abduction and flexion. The phone was strapped to the wrist and reading on the inclinometer was set to zero at 90 abducted shoulder in a lying position on a Bobath’s plinth for IR and ER. Measurements for abduction, flexion, reaching up behind the back and down behind the neck were taken in the standing position. Two undergraduate students for their respective genders in each group took the measurement. The consensus was reached by taking the mean value by each student. Students were supervised by senior trained physiotherapists. Measurements were taken before and immediately after the intervention.

### Interventions:

A senior physiotherapist trained by the principal investigator applied each following interventions to their respective group.

### Pragmatic Posterior Capsular Stretch^26^ (PPCS) group

The subject is positioned in a side-lying position. The scapula of the arm is maximally protracted and held in protraction by inserting the thumb in the axilla and fingers spiral around the spinous process of the scapula. The arm of the subject is then brought behind the back and is instructed to reach up as far as he/she can. The therapists’ other arm does the following.

- Traction is applied to the patient’s arm while the scapula is stabilised in protraction
- The patient arm is pushed gently in extension
- and medial rotation

The semi-flexed elbow provides the necessary leverage and the maneuver is performed in a way to mimic the active RUBTB. The stretch is maintained for 30 seconds combined with at least three deep breaths and each breath is held in deep inspiration for around 8–10 seconds. Three repetitions are performed.

### Serratus Anterior Stretch (SAS) group^27^

The subject is positioned in a side-lying. The shoulder for the intervention is position up and the subjected is instructed to relax. The therapist positioned himself in front of the subject. The therapist stabilizes the arm of the patient in his/her axilla and the thumb of her other hand slides on the serratus anterior under the scapula and over the digitations of the serratus anterior, and in contact with the costal surface as far as the subject can tolerate. The maneuvere attempts to separate the scapula from the ribs. Once the thumb sinks in under the scapula, the subject is asked to take a deep breath and hold for at least 8–10 seconds- Each stretch lasts for 30 seconds. Three deep breaths are taken for a single stretch. Three stretches are administered.

### Rotator Cuff Facilitation (RCF)

The subject is positioned in a supine lying and asked to flex his/her extended arm as far as he/she can. The thumb is inserted and placed over the belly of the subscapularis. Fingers of the same hand are placed over the infraspinatus muscle. A gentle sweep/effleurage/kneading through the thumb and fingers is administered over the belly of the muscles towards their attachments on the scapula simultaneously when the subject attempted to flex his/her arm at the shoulder. The subject might feel mild discomfort and the procedure is reassuring for the therapist while the therapist appreciated the increase in ROM. Infraspinatus and subscapularis are swept/kneaded simultaneously. Three repetitions of the same procedure are administered.

Subscapularis alone is swept/kneaded with arm in external rotation and the same administered to infraspinatus during arm internal rotation. The motion of the arm is solely in control of the patient and the therapist refrained himself/herself from a passive push to the arm during the intervention to avoid biases.

### Acromioclavicular Joint Mobilization group

The subject is asked to sit comfortably on a chair. The therapist positions himself behind the back of the chair. ACJ is located and the therapist places his/her thumb behind the posterior border of the clavicle. A gentle push is applied anteriorly through the thumb and posteriorly through the fingers. The therapist uses his/her other hand to cuff the upper part of the deltoid for stabilization of the shoulder/scapula. Five to ten oscillations per minute are applied three times. The applied force in anterior and posterior direction is held for 10 seconds combined with deep breathing. The subject is asked to report discomfort and the applied force is adjusted.

### Statistical analyses:

The Statistical Package for Social Science (version 21) software was used for data analyses. The independent variables were treatment groups (SAS, PPCS, RCF, ACJM). The dependent variables were flexion, abduction, IR, ER, TR, RBTB and RBTN taken before and after the intervention. Mean and standard deviation at baseline values were calculated for continuous variables and frequencies were calculated for categorical and nominal variables. Normal distribution of the isolated data for each group was determined through Kolmogorov Smirnov and Shapiro Wilk tests. Non-significant (P>0.05) results were found through both test for most of the variables. A paired t-test was used to compute significant improvement from pre-intervention measurement for each variable through each intervention.

A different variable from the baseline measurements (post-intervention minus pre-intervention) was computed for the variables of ROM. The difference in functional movement (RBTB, RBTN) was determined by subtracting the post-intervention measurements from the pre-intervention measurements since lesser values represent a greater change. This method also reduces the variability effect of the baseline values. A Kruskal Willis test was used to compare the mean ranks of each intervention for each variable.

## RESULTS

A total of 120 participants were included in equal proportion (1:1) of both genders. Equal numbers of subjects (n=30) were recruited for each intervention group. The mean age (±SD) of the participant was 21.09 (±1.79) years, height 5.47 (±0.26) feet, weight 65.82 (±13.88) kg and body mass index 23.64(±4.56) kg/m.^2^ In relation to the BMI categories, 14 (11.7%) were the underweight (<18 Kg/m^2^), 70 (58.3%) were normal limits of BMI(1 18–25 Kg/m^2^), 25 (20%) were in overweight category (25–30 Kg/m2) and 11 (9.2%) were in obese category (>30 Kg/m2). Dominant hand in 111 (92.5%) participants were right side. About the involvement of the participants in sports, 27 (22.5%) were playing cricket, 22 (18.3%) were playing volleyball, 35 (29.2%) were involved in other sports such as badminton and table tennis and 36 (30.0%) were not playing any sports but would use the shoulder in overhead household chores. Most of these participants not playing any sports were females.

All the treatment groups were balanced with respect to the number of subjects and genders. Differences in age for each treatment group were not significant. Table-I and Table-II show the descriptive statistics of all the dependent variables.

**Table-I:** Descriptive statistics of Reaching Up Behind the Back and Reaching Down Behind the Neck.

	Interventions	Pre-Intervention	Post-intervention	Difference	% Change	Cohen’s d
RUBTB	PPCS	24.13 (3.84)	17.18 (3.88)	5.09(2.21)	28.8 (11.5)	2.31
	SAS	20.84 (2.62)	15.74 (2.88)	3.24 (2.07)	24.7 (10.5)	1.56
	RCF	15.81 (6.27)	11.96 (5.03)	2.95 (2.02)	22.0 (13.9)	1.46
	ACJM	16.38 (5.23)	13.42 (5.10)	4.56 (2.78)	18.9 (12.8)	1.64
RDBTN	PPCS	24.15 (5.10)	16.71 (5.36)	7.44 (3.86)	31.0(14.9)	1.92
	SAS	23.74 (4.21)	18.34 (3.90)	5.40 (2.89)	22.5 (11.0)	1.86
	RCF	17.06 (6.49)	13.16 (6.59)	3.90 (2.87)	28.8 (21.2)	1.35
	ACJM	16.71 (4.89)	13.26 (4.64)	3.20 (1.50)	20.3 (10.8)	2.13

PPCT: Pragmatic posterior capsular stretch; SAS: Serratus Anterior stretch; RCF: Rotator cuff facilitation;

ACJM: Acromioclavicular Joint Mobilisation; RUBTB: Reaching up Behind The Back;

RDBTN: Reaching down behind the Neck; Pre-post intervention Paired t-test significant (p<0.05).

**Table-II:** Descriptive statistics for Shoulder Range of Motion.

Shoulder ROM	Interventions	Pre-intervention Mean (±SD)	Post-intervention Mean (±SD)	Difference Mean (±SD)	∆ Mean(±SD)%	Cohen’s d
Flexion	PPCS	152.40 (11.69)	167.43 (9.46)	15.03 (7.60)	10.1 (5.9)	1.97
	SAS	154.63 (9.76)	171.93 (8.90)	17.30 (8.27)	11.4 (5.9)	2.09
	RCF	158.20 (7.3)	169.40 (7.96)	11.2 (8.77)	7.2 (5.9)	1.27
	ACJM	163.80 (9.84)	170.66 (10.09)	6.32 (5.32)	4.2 (3.3)	1.18
Abduction	PPCS	151.36 (11.75)	164.26 (9.37)	12.90 (7.28)	9.2 (4.8)	1.77
	SAS	155.16 (14.69)	167.10 (17.06)	11.93 (7.91)	7.6 (5.2)	1.50
	RCF	151.508.69)	165.13 (10.58)	13.63 (8.47)	9.1 (5.9)	1.60
	ACJM	159.50 (14.60)	168.33 (10.41)	8.83 (7.22)	5.9 (5.5)	1.22
Internal Rotation	PPCS	53.56 (12.09)	64.43 (11.50)	10.86 (7.94)	23.2 (20.8)	1.36
	SAS	55.16 (12.42)	62.00 (11.18)	6.83 (6.49)	14.4 (16.9)	1.05
	RCF	66.86 (11.45)	81.96 (5.84)	13.63 (8.47)	26.3 (26.0)	1.60
	ACJM	71.46 (11.96)	79.06 (10.36)	7.60 (5.71)	11.5 (9.5)	1.33
External Rotation	PPCS	71.60 (8.64)	79.73 (9.7)	8.13 (6.61)	12.0 (9.2)	1.22
	SAS	46.33(11.81)	52.66 (14.30)	6.33 (8.70)	14.6 (21.1)	0.72
	RCF	79.23 (10.78)	91.96 (12.04)	12.73 (6.69)	16.5 (9.6)	1.90
	ACJM	78.76 (9.88)	83.83 (9.20)	5.06 (4.43)	6.7 (6.3)	1.14
Total Rotation	PPCS	125.16 (16.26)	144.16 (17.09)	19.26 (11.04)	16.0 (10.0)	1.74
	SAS	102.16 (21.20)	113.33 (23.68)	13.16 (13.54)	13.7 (15.3)	0.97
	RCF	146.10 (17.35)	173.93 (12.79)	27.83 (13.87)	20.1 (12.4)	2.00
	ACJM	150.23 (12.33)	163.13 (11.52)	12.66 (7.87)	8.6 (5.7)	1.60

PPCT: Pragmatic posterior capsular stretch; SAS: Serratus Anterior stretch; RCF: Rotator cuff facilitation;

ACJM: Acromioclavicular Joint Mobilisation; RBTB: Reaching up Behind The Back;

RBTN: Reaching down behind the Neck; Pre-post intervention Paired t-test significant (p<0.05).

All the dependent variables improved from their baseline measurement and Paired t-test statistics were highly significant (p<0.001). A very large effect size (<0.8) was observed across all the dependent variables. Shapiro-Wilk and Kolmogorov-Smirnov test for all changes in each variable were significant (p<0.05) and homogeneity could not be assumed among the four groups. A Kruskal Wallis test statics is shown in Table-III which reveals highly significant difference (p<0.001) among the means ranks of each intervention group with respect to the dependent variable except abduction (P=0.012).

**Table-III:** Kruskal Wallis test statistics for variance among the treatment groups and dependent variables of difference (∆).

Range of Motion	PPCS	SAS	RCF	ACJM	Chi- Square (χ2) df=3	P value
∆Flexion Mean Ranks	72.45	80.80	53.25	35.50	30.875	0.000
∆Abduction Mean Ranks	70.15	61.10	67.47	43.28	10.986	0.012
∆Internal Rotation Mean Ranks	66.10	46.00	78.18	51.72	15.971	0.001
∆External Rotation Mean Ranks	63.53	46.48	85.50	46.48	25.781	0.000
∆Total Rotation Mean Ranks	66.92	41.85	87.67	45.57	33.582	0.000
∆RBTB in cm Mean Rank	88.15	70.65	44.78	38.42	39.804	0.000
∆RBTN in cm ∆Mean Ranks	85.03	67.17	49.20	40.60	29.057	0.000

The relative effectiveness of each intervention for the isolated dependent variable is depicted in Table-II. The statistics in Table-II revealed greater effectiveness of PPCS on the RBTB and RBTN. SAS showed greater improvement on the Abduction. RCF was more effective in improving the rotational ROM while ACJM was useful in improving flexion and abduction movement with the least effect on the RBTB and RBTN.

## DISCUSSION

The results of this study showed that the four interventions effectively improved shoulder ROM from the baseline measurement and the effect of each intervention varied for each isolated ROM. The improvement in range of motion and functional movement in a single session of treatment is dramatic in healthy young subjects and conforms to the normative values^28^ for the age group in the current study. The interventions effectively target the areas of deficits reported as asymptotic precursors of shoulder pain.^5^, ^7^ However, promising results may have been influenced by supervised training and guidance of the principal investigator of the therapist who delivered the interventions.

It is interesting and was expected that each of the four interventions has affected a different aspect of the shoulder. The PPCS and SAS had a greater influence on the functional movements of RUBTB and RDBTN. The RCF showed greater effect on the internal and external rotation since the intervention mainly affects the subscapularis and infraspinatus which has a major role in the two movements.

The most marked effects of PPCS were on the limitations in RBTB and RBTN because the intervention principally addresses tightness in the posterior capsule as reported previously.^5^ Once the tightness was released, the limitation in abduction, flexion and rotation spontaneously improved. These findings are consistent with the findings of Rosa et al (2019) who observed that the association of posterior capsular tightness with the decreases the shoulder ROM.^10^,^26^ Similar but less clinically meaningful improvements are reported with sleeper’s stretch, crossbody stretch and modified stretch for the posterior capsular tightness.^14^,^15^ The difference is because the PPCS is more specific, passive and target the posterior capsule in a much precise manner. Furthermore, the other forms of stretches are applied at 90 degrees of flexion in which the greater tubercle of the humerus tends to translate upward and less stretching effect reaches the capsule. During the movement above 90 degrees, larger space is required for humeral head occupation and the translation is essential. Due to this phenomenon, a precise stretch is not possible and modified stretch was proposed.^14^

The SAS helps improve the timing between the lower trapezius and serratus anterior and improves the posterior-tilt and upward rotation of the scapula during the elevation of the arm. The finding of the current study reveals improvement in flexion, internal rotation, and external rotation along with the improvement in RBTB and RB TN. The opposite is reported true in patients with subacromial impingement.^29^ SAS improves the shoulder movements in a similar fashion as of PPCS. Serratus anterior is a key player in providing stability to the scapula during its movement around the rib cage. The SA dysfunction is believed to cause abnormal tension/stresses to the anterior capsular structures and increase the risk of rotator cuff impingement.^5^,^30^ In addition, timing disruption between the lower trapezius and serratus anterior has been observed in patients with subacromial impingement syndrome.^31^ The stretching described in this study may also affect the posterior capsule tightness, the mobility of the thoracic spine, rotator cuff dysfunction and ACJ stiffness which are linked with the decrease shoulder ROM. The results of the current study support the use of SAS in manual therapy protocols for restriction in shoulder joint ROM.

Rotator cuff muscles dynamically stabilize the humeral head in the glenoid cavity, and it gets dysfunctional secondary to the posterior capsular tightness, muscles force couple alteration, thoracic spine stiffness and other conditions- and therefore need to be reactivated. Subscapularis and infraspinatus (along with teres minor) in this regard are prime targets of the rotator cuff facilitation. These muscles are active in internal and external rotation at the glenohumeral joint and the findings of this study reveal a greater effect on these two motions as shown in Table-II.

The scapula is suspended from the clavicle through ACJ and requires mobility of the ACJ for its movement. The stiff ACJ, therefore hinders the movements of ACJ and affects the overhead abduction and flexion movements.^20^ The ACJM enhances the mobility at the ACJ and therefore improvements are seen in the overhead movements greater than those ROM which do not essentially require its mobility. The change in ROM produced by ACJM is less profound, yet it accentuates ACJM as an important intervention, which is needed in the final stages of rehabilitation from chronic shoulder pathology.

The participants in the current study were young and healthy college students and the results of the study might be different in professional elite athletes and older age groups with shoulder pathology. The majority of the participants were in normal and underweight BMI category. Overweight and obese category subjects are known with decrease shoulder range of motion^32^ and had little influence on the results. The study was balanced with respect to the genders and statistics may be reliable except with respect to the handedness where the dominant hand in the majority (92%) of the participants was right side, where external rotation is expected to be more at baseline.^9^ This was ruled out by incorporating the sum of external rotation and internal rotation and presented as the total rotation. The pain has inhibitory effect on the action of muscles and a decrease range of motion of shoulder is expected due to pain. Since the healthy subjects had no pain, the outcome of the intervention in subjects with shoulder main may not be duplicable. However, it is equally possible that the intervention might produce exponential improvement in shoulder range in subjects with shoulder pathology since normative ranges were achieved in healthy subjects.

This is the first study of manual therapies on the effects of pragmatic interventions and their comparison, and the results reveal a major clinical significance in improving the restricted motion of healthy subjects in a single session.

### Limitations and Recommendations

The study design is primitive, and the subjects included were healthy and young students. A trial on subjects with shoulder pathologies and restricted ROM may be needed to study the usefulness of these interventions over a longer period. Other interventions, such as thoracic spine manipulation and pectoralis minor stretching have been shown to be effective in increasing the shoulder ROM, may be combined with the current interventions to constitute a set of manual therapy intervention. The set of interventions can be used for the rehabilitation and prevention of shoulder injuries. The pragmatic intervention achieved a better range of shoulder ROM compared to the normative values^28^ of shoulder movements probably due to the younger subjects in the current study and observed the immediate effects. Further trials on prevention and shoulder rehabilitation with longer follow ups, repeated sessions and sustainability of the effects are recommended for viable recommendations.

## CONCLUSION

PPCS, SAS, RCF and ACJM are effective in increasing the shoulder range of motion. The results of the study must be interpreted cautiously since the trials were on healthy young subjects and interventions delivered by a trained and experienced trained physiotherapist.
